# The Evolving Use of Magnets in Surgery: Biomedical Considerations and a Review of Their Current Applications

**DOI:** 10.3390/bioengineering10040442

**Published:** 2023-04-01

**Authors:** William G. Lee, Lauren L. Evans, Sidney M. Johnson, Russell K. Woo

**Affiliations:** 1 Department of Surgery, Cedars-Sinai Medical Center, Los Angeles, CA 90048, USA; 2 Department of Surgery, University of Hawaii, Honolulu, HI 96822, USA

**Keywords:** review, surgery, magnet, medical, device, compression, anastomosis, bypass, stricturoplasty, minimally invasive

## Abstract

The novel use of magnetic force to optimize modern surgical techniques originated in the 1970s. Since then, magnets have been utilized as an adjunct or alternative to a wide array of existing surgical procedures, ranging from gastrointestinal to vascular surgery. As the use of magnets in surgery continues to grow, the body of knowledge on magnetic surgical devices from preclinical development to clinical implementation has expanded significantly; however, the current magnetic surgical devices can be organized based on their core function: serving as a guidance system, creating a new connection, recreating a physiologic function, or utilization of an internal–external paired magnet system. The purpose of this article is to discuss the biomedical considerations during magnetic device development and review the current surgical applications of magnetic devices.

## 1. Introduction

A magnet is a material that can produce a magnetic field based on the orientation of its atomic magnetic dipole moments. This magnetic field attracts unlike poles while repelling like poles. The resulting electromagnetic field and attractive force inherent in magnetic materials are useful properties in the development of novel surgical devices. Ever since the use of electromagnetic bougienage by Drs. Hendren and Hale for the lengthening of disconnected esophageal pouches in children with esophageal atresia, magnetic devices have been explored across the breadth of modern surgical procedures in almost every organ system (Table 1) [1].

As minimally invasive surgical (MIS) techniques, including endoscopy, laparoscopy, and robotic surgery, have allowed surgical procedures to be performed via smaller incisions (or no incisions) with improved patient outcomes (e.g., decreased hospital length of stay, faster return to activity), magnetic devices have been explored to augment existing MIS techniques and instruments [2]. With this aim, multidisciplinary and international collaboration have led to the rise in magnetic surgical device development from preclinical animal studies to the first in-human trials studying safety and feasibility [3]. This article serves to provide an overview of key biomedical considerations during device development and discuss the core functions of existing magnetic surgical devices.

**Table 1:** Historical in-human application of magnets in surgery.

Year	Organ System	Medical Indication	Use of Magnets	Citation
1975	Esophagus	Esophageal atresia	Intermittent application of electromagnetic field to metal bougies that lengthen atretic esophageal pouches	Hendren and Hale, 1975 [1]
1980	Vascular	Need for arteriovenous fistula	Intravascular paired ring magnets for side-to-side anastomosis between two blood vessels	Obora et al., 1980 [4]
1981	Colorectal	Presence of colostomy	Internal–external paired magnetic ring system for colostomy closure	Jansen et al., 1981 [5]
1989	Urology	Urethral stricture	Intraurethral miniaturized magnets for stricturoplasty	Isakov et al., 1989 [6]
1992	Small intestine	Small bowel fistula	Intraluminal paired magnets to form compression anastomosis bypassing intestinal fistula	Stepanov et al., 1992 [7]
1993	Hepatobiliary	Biliary stricture	Paired magnets to form compression anastomosis between proximal common bile duct and stomach or duodenum	Saveliev et al., 1993 [8]

## 2. Biomedical Considerations for Magnetic Surgical Devices

The utility of a magnetic material in surgical device development is dependent on its strength, durability, and mitigation of potential toxicity in the human body. The strength of a material is based on the elements that comprise it and is determined by the product of a material’s strength as well as the force required to demagnetize it (i.e., coercivity). This is known as the energy product (Gauss Oersted or Joules/m^3^) which is graded on the N grading system—increasing strength correlating with a higher grade [9]. Currently, rare-earth elements, such as N52-grade neodymium-iron-boron (Nd-Fe-B), are commonly used for industrial magnetic devices as they have the highest recorded maximum energy product (474 kJ/m^3^) [10]. This corresponds to a magnetic field of approximately 10,000 Gauss, which is 100 times stronger than a household refrigerator magnet at 100 Gauss [11]. One limitation of Nd-Fe-B magnets for industrial use is their relatively low Curie temperature, or the temperature at which a material loses its dipole alignment and subsequently its magnetism; however, the Curie temperature for Nd-Fe-B at 310 °C is well above environmental temperatures in medical use [12].

Another potential limitation is the relatively poor durability and potential toxicity of Nd-Fe-B magnets to human tissues. Nd and Fe are known to oxidize rapidly in air, leading not only to the corrosion and weakening of the magnet but also to the formation of reactive oxygen species (ROS) that have been shown to be cytotoxic to human cells [13,14]. Additionally, Nd is a brittle material that can break easily [15]. These limitations are mitigated by hermetically sealing the Nd-Fe-B magnetic core in a durable biocompatible coating. Considerations when selecting a coating include adhesion of the coating to the magnet, avoidance of magnet oxidation during the coating process, and the coating’s durability, biocompatibility, and cost [16]. Examples of biocompatible medical-grade coating materials include titanium, parylene, and gold [17,18]. During device development, the stringent testing of physical and chemical properties is regulated by the United States Food and Drug Administration (FDA) to ensure the mitigation of these risks prior to clinical implementation. Key considerations during testing include the following: type of device, duration of contact with the human body, and nature of contact with the human body (Table 2).

In addition to potential toxicity from the physical properties of Nd-Fe-B, one should consider the potential risks from exposure to a static magnetic field. Medical implants such as cardiac pacemakers or implantable cardiac defibrillators (ICD) may be at risk of interference from magnetic fields. Although the operation of implanted devices has not been shown to be adversely affected by static magnetic fields below 0.5 mT and has been proven to withstand magnetic resonance imaging (MRI), the proximity to medical implants—along with the size and strength of a magnetic surgical device—warrants consideration [19,20,21]. Theoretical carcinogenic risks from exposure to a long-term magnetic implant remain unproven given the current data on long-term MRI exposure [22].

In addition to the strength, durability, and potential toxicity of a magnetic device, a device’s intended use and location in the human body, shape, thickness, and cost are all key factors to consider during device development. More specifically, the type of tissue that the device will be in contact with (e.g., intestinal tract, biliary tract, blood vessel wall, or bone) and the method of device placement/retrieval must be considered. For example, magnetic devices that are deployed intraluminally in the foregut may be more susceptible to degradation due to their increased exposure to gastric acid and digestive enzymes, relative to devices that are implanted in bone or deployed within blood vessels. Thus, these devices may warrant additional layers of hermetic sealing and more stringent durability testing. Additionally, devices that are placed within blood vessels must consider the size, shape, and coating to facilitate feasible placement into the blood vessel—as well as the prevention of device-associated thrombus formation [23]. Thus, the intended tissue type and device environment strongly influence device design. This highlights the importance of early collaboration between clinicians and bioengineers.

The risks of magnetic surgical devices during use must also be considered during device development. Although rare, potential off-target events associated with an in situ magnetic device can lead to catastrophic morbidity and even death. This is evident in reported cases of ingested rare-earth magnets that have led to bowel fistula, perforation, sepsis, and death [24]. In order to mitigate these off-target events, devices that utilize paired systems should consider the risk of injury to the intervening tissue and the use of unidirectional ferromagnetic backplates to minimize off-target effects [25]. Considering these risks, paired systems for magnetic compression anastomosis (e.g., intestinal, biliary, vascular) also require target tissues to be apposed, without any intervening tissue/fluid, to facilitate magnet mating, avoid magnet migration, and mitigate the risk of an off-target serious adverse event. Thus, while patient selection is ultimately key in device success, optimizing tissue-specific device specifications for its intended use is also paramount.

## 3. Core Functions of Magnetic Surgical Devices

The following sections serve to discuss the core functions of existing magnetic surgical devices on the market, in addition to those of novel devices currently under investigation. They also briefly discuss the clinical uses of the individual devices introduced, but are by no means comprehensive discussions of each device (Table 3).

### 3.1. Guidance System

The ability of magnets to transmit a detectable electromagnetic field is of particular interest in the development of guidance systems for the placement of devices in the human body. This is evident in current devices that aid the placement of nasoenteric feeding tubes and peripherally inserted central venous catheters (PICCs) (Table 1).

In order to provide nutrition to critically ill patients who may be incapable of eating by mouth, small-bore nasoenteric feeding tubes are frequently placed at the bedside. Conventional placement includes blind insertion, followed by radiograph confirmation prior to the use of the tube; however, this approach can place patients at risk of the tube being placed into the airways and causing pulmonary trauma (e.g., pneumothorax), as well as cause a delay in the use of the tube for hydration, nutrition, or vital medications while the radiograph is obtained and interpreted. In addition, if the radiograph is misinterpreted, tube feeding into the lungs can lead to catastrophic morbidity and even death. An alternative approach uses a stylet with an electromagnetic tip inside of the feeding tube during placement that transmits a signal to an external receiver unit placed on the patient’s epigastric region [26]. This allows for the real-time visualization of the path of the tip of the tube to avoid placement into the lung, as well as for the confirmation of the correct placement in the stomach or small intestine without requiring radiograph confirmation. This has been shown to reduce the risk of pulmonary complications, such as feeding into the lungs or pneumothorax, while decreasing the delay in tube feeding initiation [27]. Additionally, this has reduced the burden on radiology resources and has the potential to lead to a cost avoidance of USD 346,000 over a 2-year period [28].

Similar to the placement of feeding tubes, the detection of an internal magnet for real-time guidance is also being used for PICC placement. PICCs are catheters that provide central venous access for medications, parenteral nutrition, repeated blood sampling, and invasive hemodynamic monitoring. These specialized catheters are commonly placed at the bedside under blind insertion with radiographic confirmation or in the radiology suite under live fluoroscopic guidance. While the former approach introduces an increased risk of malposition or cardiovascular injury that can result in arrhythmia or cardiac tamponade, the latter introduces increased radiation exposure, higher cost, and limited availability in resource-limited settings [31]. The magnetic approach uses an inner stylet with a magnetic tip that is detected by an external sensor placed on a patient’s sternum, providing real-time tracking of the catheter’s path, which has decreased rates of malposition and radiation exposure [29,30].

The potential of intravascular magnets in guiding the formation of arteriovenous fistulas (AVFs) in patients requiring long-term hemodialysis is being explored. AVFs continue to be created via conventional open surgery connecting an extremity artery to a vein; however, specialized centers are now exploring the ability to create AVFs in the radiology suite via percutaneous means. Specialized catheters with magnetic tips are inserted into the artery and vein of interest, which guide the catheters to the area of interest and align the vessels together, allowing a radiofrequency electrode to weld the vessels together to form a fistula [32]. Although not yet widely available, this introduces a novel use of paired magnets to guide a novel minimally invasive approach to AVF creation. Thus, existing magnetic surgical devices have improved conventional approaches by minimizing radiation exposure as well as the use of radiology resources, improving procedural accuracy, and creating a novel minimally invasive approach for a common vascular operation.

### 3.2. Magnetic Compression Anastomosis to Create New Connections

New connections can be created between two hollow organs (e.g., the esophagus, stomach, small intestine, colon, and bile duct) to bypass an obstructed segment of the gastrointestinal (GI) tract due to atresia, stricture, or mass—or to bypass the large absorptive surface of the small intestine (e.g., bariatric bypass surgery). Currently, open or laparoscopic approaches to the creation of an anastomosis are performed with either surgical staplers or hand-sutured methods; however, the use of compression to create an equally robust anastomosis resistant to anastomotic leak would create an approach that could further minimize the invasiveness of this procedure by decreasing or eliminating the need for incisions. This approach also has the potential to create an anastomosis in patients that require one to restore GI tract continuity, but that have medical or anatomic comorbidities that may prohibit conventional surgical methods [56]. For example, in patients with an obstructing gastric cancer and a history of multiple intra-abdominal operations or radiation therapy, subsequent dense adhesions may prohibit a safe palliative gastrointestinal bypass. Thus, an endoluminal magnetic approach would allow these high-risk surgical patients to receive treatment and avoid a permanent proximal gastrointestinal diversion.

The modern use of compressive force to create an anastomosis re-emerged in the 1980s with the creation of the biofragmentable bowel anastomosis ring (BAR) and compression anastomotic clips [110,111,112,113,114]. More recently, the use of paired magnets to create magnetic compression anastomoses (MCAs) has also been shown to form robust anastomoses [53]. Magnets are placed in the organs of desired connection via endoscopic- or laparoscopic-assisted approaches. Magnets are guided to mate, and once the anastomosis is fully formed the magnets unite and are excreted or retrieved endoscopically [36,39,50].

**Figure 1:**
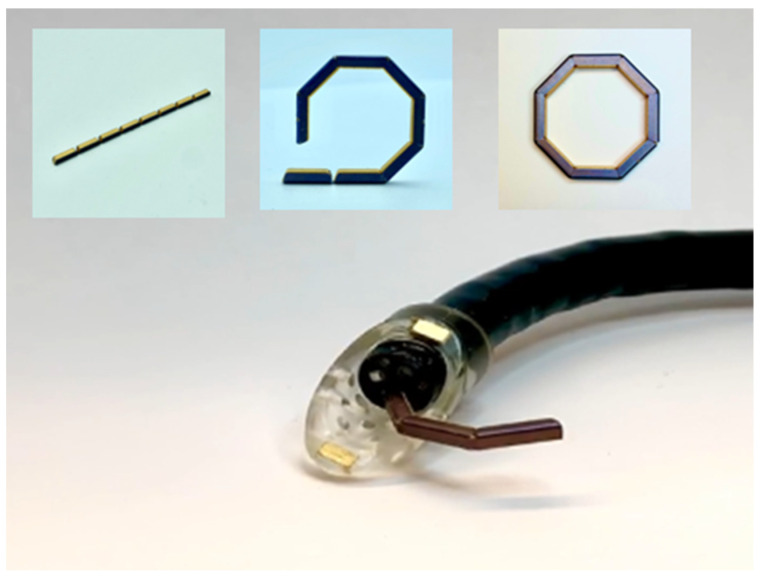
GI Windows Surgical^TM^ self-forming magnetic rings that can be delivered through an endoscope into the target segments of the bowel. Magnetics rings auto-align and mate—leading to the formation of a magnetic compression anastomosis. (Permission for use granted by GI Windows Surgical, West Bridgewater, MA, USA.)

**Figure 2:**
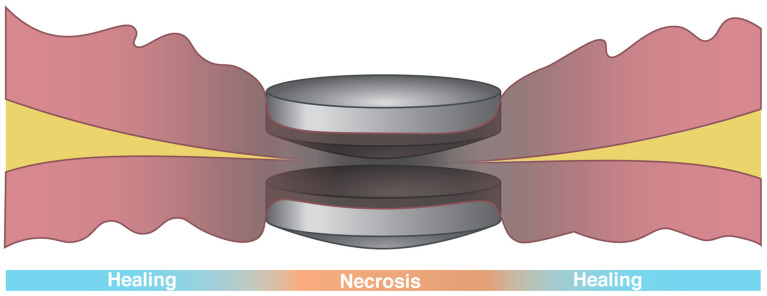
Connect-EA^TM^ device composed of two N52-grade magnets with a unique concave–convex surface that produces a pressure gradient in the intervening tissue compressed between the magnets. The maximal pressure in the center creates tissue necrosis, while allowing for tissue healing and epithelialization at the periphery, which forms a robust anastomosis with decreased stricture formation [53]. (Permission for use granted by Myka Labs, San Francisco, CA, USA.)

Various paired magnet systems have been described in the literature (Table 3). Magnet configurations include spherical, circular discoid (flat or concave–convex), circular-ring-shaped, with varying inner diameter sizes, cylindrical, and bullet-shaped [50]. The magnetic cores of Nd-Fe-B or samarium-cobalt are commonly described, with coating methods varying between being single-layer (e.g., titanium oxide, polycarbonate) and multi-layer (e.g., inner nickel-copper-nickel coating, outer gold-parylene C coating for biocompatibility) [53]. Additional considerations during the development of MCA devices include how to best place the magnets into organs of interest (e.g., endoscopy, laparoscopy, and percutaneous) and the effects of magnet size/shape on magnet delivery as well as apposition, mating force, anastomosis formation, and the risk of postoperative complications (e.g., anastomotic leak, stricture). Prior to in-human use, extensive preclinical testing on device placement, biocompatibility, compressive force, and burst pressure performance is often performed [34,36,37,38,39,40,43,115]. Burst pressure is used to test anastomotic resistance to leakage with maximal observed burst pressures in MCAs consistently being > 100 mmHg, which is above the physiologic intraluminal pressure [53]. Continued research aims to optimize magnet delivery via completely endoscopic means (i.e., incisionless surgery) (Figure 1) while improving anastomotic outcomes by reducing stricture rates (Figure 2) [45,55,56].

The miniaturization of MCA device systems has also broadened their applicability to treat many pediatric conditions. A key example of this is in infants with esophageal atresia (EA)—a congenital malformation resulting in two separate non-communicating pouches that require a surgical anastomosis in order to achieve esophageal continuity and allow for nutrition via the mouth [116]; however, the current surgical options are sometimes associated with significant morbidity (e.g., musculoskeletal deformity, anastomotic leak or stricture, or vocal cord paresis)—the rates for which have remained relatively constant over the past 80 years [117]. Thus, the MCA devices currently used for EA repair utilize paired magnets that are placed into apposing pouches—one via the mouth and one via the stomach—and mated to create a completely endoscopic anastomosis. While the widespread adoption of this technique has been limited by early experience, observing the prohibitive rates of anastomotic strictures [54], newer devices with wider mating surfaces and unique mating geometries aim to solve this problem [53,55,56,118] (Figure 2).

Examples of in-human use of MCA include the following:

- Esophago-esophageal anastomoses of two disconnected esophageal ends in children with esophageal atresia [49,51,52,54,55,56];
- Gastroenteric or intestinal anastomoses to restore gastrointestinal (GI) tract continuity [35,42];
- Uretero-ileal anastomoses for urinary diversions in patients with neurogenic bladders [42];
- Colorectal anastomoses of disconnected colons and rectums in children with rectal atresia [41];
- Intestinal anastomoses for enteral bypasses/diversions with comparable weight loss and decrease in hemoglobin A1c, fasting blood glucose, and use of medications/insulin for diabetes [44,45];
- Stricturoplasties, or intraluminal resections of obstructing strictures, to restore GI tract continuity;
- Small bowel non-anastomotic strictures [63];
- Anastomotic strictures after esophagectomy for esophageal cancer [64], esophageal atresia repair [61,62], and colon resections for colorectal cancer [66];
- Bilioenteric (the bile duct to the small intestine) anastomotic ischemic strictures after liver transplant or a bile duct injury [57,58,59,60].

Cardiac and vascular surgeons are also utilizing paired magnet systems to create new connections between two arteries. Currently, the majority of vascular anastomoses are hand-sewn; however, in contrast to MCAs, magnetic vascular ports (MVPs) are permanent devices that are deployed within arteriotomies to form a vascular port opening on each vessel of interest. Ports are then mated to form anastomoses [69]. This method has been utilized most frequently for the minimally invasive direct coronary artery bypass (MIDCAB) procedure to connect the left internal mammary artery (LIMA) on the chest wall to the left anterior descending (LAD) artery on the heart [69]. In-human trials have demonstrated decreased anastomotic and total procedure times, with no device-related adverse events and favorable patency rates at 6-month follow-ups [68,70,72,73,74,76,119]. Preclinical animal studies are also being used to explore the use of MCAs in the creation of vascular anastomoses, but these have not progressed to in-human trials at this point [120,121,122,123].

### 3.3. Recreating a Physiologic Function

The dysfunction of muscular sphincter complexes can lead to gastroesophageal reflux disease (GERD; lower esophageal sphincter) or incontinence (fecal; anal sphincter complex). Anti-reflux procedures for the treatment of medically refractory GERD, such as a Nissen fundoplication, can lead to abdominal bloating, inability to vomit, and persistent dysphagia [124]. Additionally, there are multiple surgical options for fecal incontinence, such as sacral nerve stimulation and artificial sphincter balloon implants, but the adoption of one has been limited by suboptimal outcomes [125]. Magnetic sphincter augmentation has the potential to recreate physiologic sphincter function. Current magnetic devices consist of small titanium-coated beads with Nd-Fe-B magnetic cores interlinked with titanium wires to form a flexible expandable ring (Table 3) [87]. This configuration allows for opening with increased dynamic pressure, but maintains a closed position at lower passive pressures, preventing reflux or incontinence. Devices are sized to the external diameter of the esophagus (e.g., GERD) or the anal canal (e.g., fecal incontinence) without compressing the underlying muscle [79,87]. Magnetic sphincter devices for GERD have demonstrated safety and the normalization of esophageal acid exposure (i.e., reflux), reduced or discontinued reflux medication use (i.e., proton pump inhibitors), and improved quality of life [78,80,81,83,84,85]. Magnetic devices for fecal incontinence have also shown improvements in incontinence severity and quality of life, with comparable outcomes to sacral nerve stimulation, but their adoption has been limited due to reports of device-related infection and perineal pain [88,89,126,127,128,129]. Magnetic sphincter augmentation is also being explored for urinary incontinence in the preclinical phase [90]. Thus, magnetically augmented sphincters are currently being used to recreate physiologic sphincter function, while also having the potential to be reversible (with device explantation).

### 3.4. Use of Internal–External Paired Magnet Systems

Systems that use an internal magnet guided by or acted upon by an external magnet have a variety of surgical applications, ranging from the surgical treatment of congenital disorders (e.g., scoliosis, pectus excavatum) to improving organ retraction during laparoscopic surgery (Table 3). The surgical management of early onset scoliosis employs dynamic instrumentation to allow for continued longitudinal growth. Traditional growing rods require repeated surgeries to lengthen the rods as a child grows. Magnetic growing rod (MGR) systems use a similar system with single or dual implanted titanium spinal rods fixed to the spine cranially and caudally [130]; however, as a child grows, an external magnetic device can be used to rotate the rod’s internal actuator, which lengthens the rod without the need for repeated surgeries [103]. In addition to a reduction in operations, the MGR system has also led to subsequent long-term cost savings, decreased infection rates, and similar rates of implant failure (e.g., rod or foundation failure) [104,106,107,108,109,131].

**Figure 3:**
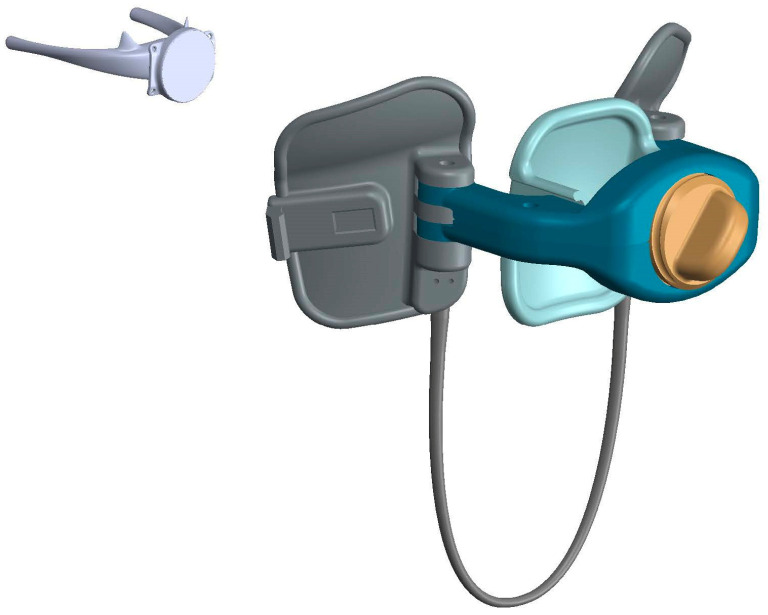
The magnetic apnea prevention (Mag-Nap) system utilizes an N52-grade neodymium-iron-boron magnet encased in titanium with a ferromagnetic directional back plate that is implanted onto the hyoid bone (**left**). The custom-fitted removable external neck accessory (**right**) to be worn during sleep contains a second magnet that attracts the implanted hyoid magnet forward to keep the airway open [102]. (Permission for use granted by Mag-Nap Inc., San Francisco, CA, USA).

The attractive magnetic force of an internal–external paired magnet system is also being employed to gradually remodel musculoskeletal deformities. Pectus excavatum is a congenital deformation of the cartilages that connect the ribs to the sternum that pushes the sternum inward and can cause the compression of the heart. Conventional repairs involve major surgical reconstruction. The modified Ravitch procedure requires the open removal of the abnormal cartilages, the fracturing of the sternum, and fixation into a satisfactory alignment. The less invasive Nuss procedure uses smaller incisions to place a titanium metal bar within the chest and behind the sternum to gradually remodel the chest wall over a 2-year period; however, both procedures are associated with a serious risk of injury to the heart, lungs, or major blood vessels, as well as significant postoperative pain. The Nuss procedure also requires a second procedure to remove the titanium bar after te remodeling of the chest wall is complete. The magnetic approach implants an internal Nd-Fe-B magnet with a ferromagnetic focusing plate encapsulated in a low-profile titanium shell to the anterior sternum [25,99]. The internal magnet is mated with a second magnet housed in an external polypropylene custom-fitted anterior chest wall brace [25]. This approach has been shown to decrease surgical risk while improving chest wall deformation and decreasing overall cost [100,101]; however, this technology has seen limited adoption due to major improvements in postoperative pain control with the advent of intercostal nerve cryoablation [132]. A similar use of an internal–external paired magnet system is also being studied in obstructive sleep apnea treatment—as an alternative to continuous positive airway pressure (CPAP)—by advancing the hyoid bone forward to maintain airway patency during sleep (Figure 3) [102,133]. Other surgical applications of an internal–external paired magnetic system include the use of internal detachable magnetic graspers manipulated by external magnets to aid in organ retraction during laparoscopic gallbladder surgery and bariatric surgery to decrease the number of incisions (Table 3) [91,92,93,95,96,134]. These multiple uses highlight the potential and versatility of magnetic surgical systems that can manipulate internal tissues via an external source.

## 4. Conclusions

The use of magnetic devices in surgery is an actively growing field with various systems on the market or in preclinical development. Multidisciplinary collaboration during device development is recommended due to the combination of biomedical engineering and surgical considerations. Existing magnetic surgical devices can be used to guide the placement of catheters and tubes, create new connections in the GI tract as well as blood vessels, recreate physiologic sphincter function, and manipulate internal tissues via an external magnet. This versatility highlights the potential of magnetic devices to improve existing surgical techniques and minimally invasive approaches.

## Figures and Tables

**Table 2:** FDA framework for the evaluation of biocompatibility in device development.

Contact Duration	Type of Device	Nature of Body Contact	Recommended Endpoint Testing
Limited contact (≤24 h)	Surface device	Intact skin	C, S, and I
		Mucosal membrane	C, S, and I
		Breached or compromised surface	C, S, I, Sy, and P
	External communicating device	Blood path, indirect	C, S, I, Sy, P, and H
		Tissue/bone/dentin	C, S, I, Sy, and P
		Circulating blood	C, S, I, Sy, P, G, and H
	Implant device	Tissue/bone	C, S, I, Sy, and P
		Blood	C, S, I, Sy, P, G, Im, and H
Prolonged contact (>24 h to ≤30 days)	Surface device	Intact skin	C, S, and I
		Mucosal membrane	C, S, I, Sy, P, Sub, and Im
		Breached or compromised surface	C, S, I, Sy, P, Sub, and Im
	External communicating device	Blood path, indirect	C, S, I, Sy, P, Sub, and H
		Tissue/bone/dentin	C, S, I, Sy, P, Sub, G, and Im
		Circulating blood	C, S, I, Sy, P, Sub, G, Im, and H
	Implant device	Tissue/bone	C, S, I, Sy, P, Sub, G, and Im
		Blood	C, S, I, Sy, P, Sub, G, Im, and H
Long-term/ permanent contact (>30 days)	Surface device	Intact skin	C, S, and I
		Mucosal membrane	C, S, I, Sy, P, Sub, G, Im, and Ct
		Breached or compromised surface	C, S, I, Sy, P, Sub, G, Im, Ct, and Car
	External communicating device	Blood path, indirect	C, S, I, Sy, P, Sub, G, Im, Ct, and Car
		Tissue/bone/dentin	C, S, I, Sy, P, Sub, G, Im, Ct, Car, and H
		Circulating blood	C, S, I, Sy, P, Sub, G, Im, Ct, and Car
	Implant device	Tissue/bone	C, S, I, Sy, P, Sub, G, Im, Ct, and Car
		Blood	C, S, I, Sy, P, Sub, G, Im, Ct, Car, and H

C (cytotoxicity), S (sensitization), I (irritation or intracutaneous reactivity), Sy (acute systemic toxicity), P (material-mediated pyrogenicity), H (hemocompatibility), G (genotoxicity), Im (implantation), Sub (subacute/subchronic toxicity), Ct (chronic toxicity), and Car (carcinogenicity).

**Table 3:** Published surgical applications of magnetic devices.

Core Function	Type of Magnet System	Use of Magnet System	Example of Devices Approved for Humanitarian or Commercial Use	Published Studies
GS	Electromagnetic tube/catheter tip and external receiver unit	Real-time transmission of nasoenteric tube location during placement	CORTRAK Enteral Access System (Avanos Medical, Inc., Alpharetta, Georgia, USA)	Mathus-Vliegen 2010 [26], Smithard 2015 [27], an McCutcheon 2017 [28]
		Real-time transmission of peripherally inserted central venous catheter (PICC) location during placement	Sherlock 3CG Tip Confirmation System (Becton, Dickinson and Company, Franklin Lakes, NJ, USA)	Tomaszewski 2017 [29], Mack 2020 [30], and Sone 2020 [31]
	Paired intravascular magnetic catheter tips	Mating of catheter tips aligns as well as holds an artery and vein together for percutaneous arteriovenous fistula creation	everlinQ endoAVF System (Becton, Dickinson and Company, Franklin Lakes, NJ, USA)	Lok 2017 [32]
NC	Magnetic compression anastomosis between two paired intraluminal magnets (spherical, discoid, ring, and cylindrical)	Connecting two small intestine segments		Xu 2015 [33]
		Connecting two enteric segments (e.g., stomach, small intestine, and colon)	Magnamosis Magnetic Compression Anastomosis Device (Myka Labs, UCSF Surgical Innovations, San Francisco, CA, USA)	Cope 1995 [34], Chopita 2005 [35], Jamshidi 2009 [36], Myers 2010 [37], Pichakron 2011 [38], Gonzales 2012 [39], Wall 2013 [40], Russell 2014 [41], and Graves 2017 [42]
		Connecting the proximal intestine to the distal intestine to create a bypass channel (i.e., bariatric surgery)	Self-Forming Magnetic Anastomosis Device (GI Windows Surgical, West Bridgewater, MA, USA)	Ryou 2016 [43], Machytka 2017 [44], Schlottman 2021 [45], Gumustop 2022 [46], and Ore 2022 [47,48]
		Connecting the proximal and distal esophageal pouches in esophageal atresia (congenital disorder)	Magnamosis Connect-EA (Myka Labs, UCSF Surgical Innovations, San Francisco, CA, USA); Flourish Pediatric Esophageal Atresia Device (Cook Medical, Bloomington, IN, USA)	Zaritzky 2009 [49], Zaritzky 2014 [50], Dorman 2016 [51], Slater 2019 [52], Muensterer 2020 [53], Wolfe 2020 [54], Muensterer 2021 [55], and Evans 2022 [56]
		Connecting the bile duct to the stomach or small intestine to bypass bile duct stricture		Mimuro 2003 [57], Muraoka 2005 [58], Matsuno 2009 [59], and Jang 2020 [60]
		Resecting a strictured esophagus, small intestine, bile duct, or colon to allow luminal contents to pass through		Takamizawa 2007 [61], Woo 2017 [62], Kamada 2020 [63], Isozaki 2020 [64], Liu 2020 [65], Kılıç 2020 [66], and Liu 2022 [67]
	Paired intravascular magnetic ports	Creation of anastomosis between two blood vessels (e.g., coronary artery bypass surgery)	Magnetic Vascular Positioner (MVP) Series 6000 Distal Anastomosis System (Ventrica, Inc., Fremont, CA, USA)	Falk 2003 [68], Klima 2003 [69], Klima 2004 [70], Wong 2004 [71], Athanasiou 2004 [72], Falk 2005 [73], Vicol 2005 [74], Klima 2006 [75], Vicol 2006 [76], and Charitou 2006 [77]
PHYS	Magnetic beads interlinked with titanium wires to form a flexible ring	Placed around the distal esophagus to recreate a physiologic lower esophageal sphincter in gastroesophageal reflux disease (GERD)	LINX (Torax Medical, Inc., Shoreview, MN, USA)	Lipham 2012 [78], Ganz 2013 [79], Bonavina 2013 [80], Smith 2014 [81], Bauer 2015 [82], Saino 2015 [83], Aiolfi 2018 [84], and Bell 2020 [85]
		Placed around the external anal sphincter to recreate physiologic sphincter function in fecal incontinence	FENIX Continence Restoration System (Torax Medical, Inc., Shoreview, MN, USA)	Bortolotti 2008 [86], Lehur 2010 [87], Barussaud 2013 [88], and Jayne 2021 [89]
	Paired flat magnets	Placed anterior and posterior to the urethra to recreate urethral resistance in urinary incontinence		Ali-El-Dein 2000 [90]
IE	Detachable internal magnetic grasper controlled by an external magnet	Retraction of the gallbladder during cholecystectomy	Levita Magnetic Surgical System (Levita Magnetics, Inc., Menlo Park, CA, USA)	Dominguez 2009 [91], Rivas 2018 [92], and Haskins 2018 [93]
		Retraction of the liver, stomach, or omentum during bariatric surgery		Morales-Conde 2013 [94], Rahman 2017 [95], and Davis 2019 [96]
	Internal ureteral stent with a distal magnetic tip	Bedside removal of a stent by mating an internal stent tip with an introduced urethral magnetic catheter retrieval device	Magnetic Blackstar (Urovision-Urotech, Achenmuhle, Germany)	Rassweiler 2017 [97], Sevcenco 2018 [98]
	Implanted magnet and custom-fitted external brace with a paired magnet	Gradual chest wall remodeling in the pectus excavatum (congenital disorder)	Magnetic Mini-Mover (3MP): Magnimplant and Magnatract (Hayes Manufacturing, Sunnyvale, CA and Hantel Technologies, Hayward, CA, USA)	Harrison 2007 [25], Harrison 2010 [99], Harrison 2012 [100], and Graves 2017 [101]
		Hyoid bone advancement to maintain airway patency during sleep in obstructive sleep apnea (OSA)	Magnetic Apnea Prevention Device (Mag-Nap) (Mag-Nap, Inc., UCSF Surgical Innovations, San Francisco, CA, USA)	Rosenbluth 2011 [102]
	Implanted distractable spinal rods with a magnetic lengthening mechanism driven by external magnetic remote control	Non-invasive spinal adjustment allows for the growth of a child with early onset scoliosis (congenital disorder)	MAGEC System (NuVasive, Inc., San Diego, CA, USA)	Cheung 2012 [103], Jenks 2014 [104], Lorenz 2017 [105], Subramanian 2018 [106], Oetgen 2019 [107], Harshavardhana 2019 [108], and Guan 2020 [109]

GS (guidance system), NC (creating new connection), PHYS (recreating physiologic function), IE (internal–external paired system).

## Data Availability

No new data were created or analyzed in this study. Data sharing is not applicable to this article.
